# Gut Microbiota Prevents Sugar Alcohol-Induced Diarrhea

**DOI:** 10.3390/nu13062029

**Published:** 2021-06-12

**Authors:** Kouya Hattori, Masahiro Akiyama, Natsumi Seki, Kyosuke Yakabe, Koji Hase, Yun-Gi Kim

**Affiliations:** 1 Research Center for Drug Discovery, Faculty of Pharmacy and Graduate School of Pharmaceutical Sciences, Keio University, Tokyo 105-8512, Japan; kouya19950425@keio.jp (K.H.); akiyama.masahiro@keio.jp (M.A.); natsumisoshiru826@keio.jp (N.S.); kkkeeoosuyi@keio.jp (K.Y.); 2 Department of Biochemistry, Faculty of Pharmacy and Graduate School of Pharmaceutical Sciences, Keio University, Tokyo 105-8512, Japan; hase-kj@pha.keio.ac.jp

**Keywords:** gut microbiota, sugar alcohol, diarrhea

## Abstract

While poorly-absorbed sugar alcohols such as sorbitol are widely used as sweeteners, they may induce diarrhea in some individuals. However, the factors which determine an individual’s susceptibility to sugar alcohol-induced diarrhea remain unknown. Here, we show that specific gut bacteria are involved in the suppression of sorbitol-induced diarrhea. Based on 16S rDNA analysis, the abundance of Enterobacteriaceae bacteria increased in response to sorbitol consumption. We found that *Escherichia coli* of the family Enterobacteriaceae degraded sorbitol and suppressed sorbitol-induced diarrhea. Finally, we showed that the metabolism of sorbitol by the *E. coli* sugar phosphotransferase system helped suppress sorbitol-induced diarrhea. Therefore, gut microbiota prevented sugar alcohol-induced diarrhea by degrading sorbitol in the gut. The identification of the gut bacteria which respond to and degrade sugar alcohols in the intestine has implications for microbiome science, processed food science, and public health.

## 1. Introduction

Sugar alcohols such as sorbitol, mannitol, and xylitol are naturally present in some fruits, vegetables, and mushrooms but are classified as artificial sweeteners since they can be industrially produced by reducing saccharides such as glucose [1,2]. These sugar alcohols have a low caloric content due to poor absorption in the small intestine and are thus frequently used as sweeteners in “sugar-free” candy, chewing gum, and beverages [3]. Because of their stability and resistance to heat, they are also used for a wide variety of other purposes, including as food moisturizing agents, preservatives, and excipients. However, ingestion of these poorly-absorbed sugar alcohols may lead to gastrointestinal symptoms in some people and have laxative effects. For example, it has been reported that the excessive use of chewing gum containing sorbitol may cause symptoms such as diarrhea, weight loss, and bloating [4,5]. The main cause of the diarrhea is thought to be the accumulation of poorly-absorbed sugar alcohols in the colon, where they increase colonic osmotic pressure and prevent water absorption [6]. Interestingly, susceptibility to sugar alcohol–induced diarrhea is known to vary among individuals [6]. However, the mechanisms underlying these effects remain unknown.

Poorly-absorbed carbohydrates can be a source of energy for some bacteria residing in the gut [7]. The human gut contains approximately 40 trillion bacteria that form complex communities of which the composition varies among individuals due to differences in diet, lifestyle, and antibiotic use [8,9]. Consumption of poorly-absorbed carbohydrates is one factor that can alter the composition of the gut microbiota. Because the carbohydrates preferred by gut bacteria vary from species to species, the ingestion of poorly-absorbed carbohydrates with different chemical structures can promote the development of a distinctive gut flora [10]. Additionally, sugar alcohols like sorbitol, which are a type of poorly-absorbed carbohydrate, are known to be degraded by certain bacteria [11,12]. However, the relationship between the consumption of poorly-absorbed sugar alcohols and their biological effects, such as osmotic diarrhea and gut bacteria makeup, remains poorly understood.

We hypothesized that sugar alcohol-degrading bacteria exist in the intestine and also that susceptibility to sugar alcohol-induced diarrhea depends on the amount of these bacteria being present. When the intestine contains low amounts of sugar alcohol–degrading bacteria, sugar alcohols accumulate, which leads to increased osmotic pressure and diarrhea. In the current study, we investigated the relationship between sugar alcohol-induced diarrhea and gut microbiota in an effort to identify the bacteria responsible for the prevention of diarrhea symptoms.

## 2. Materials and Methods

### 2.1. Mice

Male C57BL/6 mice were purchased from the Sankyo Labo Service Corporation (Tokyo, Japan) and were kept under conventional conditions. Male MCH (ICR) mice, germ-free (GF) male ICR mice, and ICR-derived inbred strain IQI mice were purchased from CLEA Japan, Inc. (Tokyo, Japan). GF male MCH and IQI mice were maintained in vinyl isolators. Male MCH mice were kept under specific pathogen-free (SPF) conditions. These mice were fed γ-ray-sterilized AIN-93G (Oriental Yeast, Tokyo, Japan). In order to evaluate the effect of sugar alcohol on diarrhea, mice were administered water drinking water containing 5% or 10% sorbitol, or 5% mannitol for 4 days. For experiments involving antibiotics, mice were administered drinking water containing ampicillin (1 g/L), streptomycin (5 g/L), erythromycin (200 mg/L), or vancomycin (250 mg/L). For gnotobiotic experiments, GF male ICR or IQI mice (obtained from CLEA Japan) were bred in vinyl isolators and fed γ-ray-sterilized CMF. During the experiment, GF mice were fed a γ-ray-sterilized AIN-93G.

All mice were housed under 21–22 °C with a 12 h alternating light–dark cycle at the animal facilities of Faculty of Pharmacy, Keio University (Tokyo, Japan). All animal experiments were performed according to the Institutional Guidelines for the Care and Use of Laboratory Animals in Research and were approved by the local ethics committees at Keio University.

### 2.2. Bacteria

Bacteria isolated from mouse feces and strains purchased from the Japan Collection of Microorganisms (JCM, Ibaraki, Japan) were used in the study. Wild-type (WT) and gene mutants (*srlA*-, *srlB*-, *srlE*-, or *srlD*) of *E. coli* K-12 were purchased from the National Bio Resource Project (NBRP, Shizuoka, Japan).

### 2.3. Colonization of Germ-Free Mice

*Escherichia coli* and *Proteus mirabilis* isolated from the stool of C57BL/6 mice, or WT and *srlD* mutant of *E. coli* K-12 were used for the experiments. GF male IQI or ICR mice were colonized with 200 µL of bacterial culture via oral gavage. 

### 2.4. Evaluation of Diarrhea Symptoms

The severity of diarrhea was evaluated based on body weight change and fecal water content after the sugar alcohol treatment. Body weight change was recorded daily in the morning. The collected feces were weighed, dried overnight in an incubator, and re-weighed. The fecal water content was calculated from the weight before drying and the weight after drying.

### 2.5. 16S rRNA Gene Sequencing and Analysis 

Approximately 200 mg of each stool sample was transferred into 2 mL tubes containing 0.1 mm zirconia/silica beads and 3.0 mm zirconia beads. The stool samples were homogenized for 10 min in a Shake Master Neo cell disruption device (Biomedical Sciences, Tokyo, Japan) after the addition of 540 µL of SLX–Mlus Buffer from an E.Z.N.A. Stool DNA Kit (Omega Bio-tek, Norcross, GA, USA). After homogenization, 60 µL of DS buffer and 20 µL of Proteinase K solution from the E.Z.N.A. Stool DNA Kit were added. The samples were then incubated at 70 °C for 10 min and at 95 °C for 5 min. SP2 buffer from the E.Z.N.A. Stool DNA Kit was then added and the samples were incubated on ice for 5 min. The samples were centrifuged at 13,000× *g* for 5 min and DNA was extracted from 200 µL of the supernatant using a magLEAD 12 gC nucleic acid extraction instrument (Precision System Science, Chiba, Japan) with a MagLEAD Consumable Kit and MagDEA^®^Dx SV. The extracted genomic DNA was resuspended at 5 ng/μL in 10 mM Tris-HCl buffer. The DNA was used to prepare 16S rRNA gene libraries in accordance with the protocol described in an Illumina technical note (https://support.illumina.com/documents/documentation/chemistry_documentation/16s/16s-metagenomic-library-prep-guide-15044223-b.pdf (accessed on 9 May 2019). Briefly, each DNA sample was amplified by polymerase chain reaction (PCR) using KAPA HiFi HS ReadyMix (Nippon Genetics, Tokyo, Japan) and primers specific for variable regions 3 and 4 of the 16S rRNA gene. The PCR products were purified using AMPure XP Beads (Beckman Coulter, Brea, CA, USA) and adapters added by PCR using a Nextera XT Index Kit. The libraries were further purified using AMPure XP Beads, diluted to 10 nM with 10 mM Tris–HCl buffer, and pooled. The pooled samples were sequenced using a MiSeq System (Illumina, San Diego, CA, USA) with a 2 × 300-base-pair protocol. Sequences were analyzed using QIIME (v. 1.9.1) software (http://qiime.org, accessed on 10 June 2021) [13]. Reads with an average quality value < 20 were excluded using Trimmomatic (v. 0.36) software (http://www.usadellab.org/cms/?page=trimmomatic, accessed on 10 June 2021) [14]. Paired-end sequences were joined using a Fastq-join tool in the EA-Utils software package (https://expressionanalysis.github.io/ea-utils/, https://openbioinformaticsjournal.com/VOLUME/7/PAGE/1/, accessed on 10 June 2021). High-quality sequences (11,000) were randomly chosen from the quality filter-passed sequences that were obtained for each sample. After trimming both primer sequences using cutadapt (https://doi.org/10.14806/ej.17.1.200, accessed on 10 June 2021), chimeras were detected using the USEARCH de novo method. The sequences were assigned to operational taxonomic units (OTUs) using the UCLUST algorithm with a sequence identity threshold of 96%. Taxonomic assignments for each OTU were made by similarity searching against publicly available 16S sequences (RDP v. 10.27 (The Center for Microbial Ecology, East Lansing, Michigan, US) and CORE update 2 September 2012) and NCBI genome databases using the blastn program available from the local Basic Local Alignment Search Tool (BLAST). The data were simplified to 5000–10,000 sequences per sample using rarefaction curves, and the relative abundances of the community members were determined using the rarefied data. The analysis was performed as described in a previous report [15]. Principal coordinate analysis (PCoA) was used to analyze the beta diversity using an unweighted UniFrac metric calculated by QIIME. Permutational multivariate analysis of the variance (PERMANOVA) test was performed using the vegan package (v 2.7) (https://github.com/vegandevs/vegan, accessed on 10 June 2021) in R.

### 2.6. DNA Extraction and Quantitative PCR (qPCR)

Bacterial DNA was isolated from 20 mg of fecal samples (or 200 µL of standard culture). Extraction and quantification of the bacteria DNA were conducted as described in a previous report [16]. Briefly, a 20-fold diluted fecal sample (200 µL) was mixed with 300 μL of extraction buffer (100 mM Tris–HCl, 40 mM EDTA, 1.7% SDS, pH 9.0), 500 µL of buffer-saturated phenol, and 300 mg of glass beads. The mixture was vortexed vigorously for 15 s at 5000 rpm 3 times using a Precellys24 homogenizer (M&S Instruments Inc., Osaka, Japan). After centrifugation at 14,000× *g* for 5 min, 400 µL of the supernatant was collected. Phenol–chloroform extractions were subsequently performed and 250 µL of the supernatant was subjected to isopropanol precipitation. Finally, the DNA was suspended in 1 mL of TE buffer.

The total number of bacteria in the feces was analyzed by qPCR using universal primers. PCR amplification and detection were performed using a CFX-96 Real-Time system (Bio-Rad, Hercules, CA, USA). Each reaction mixture (20 µL) consisted of 10 µL of SYBR premix Ex Taq II (Takara, Shiga, Japan), 0.4 µL of each primer (10 µM), 2 µL of DNA template, and 7.2 µL of distilled water. The amplification profile consisted of one cycle at 95 °C for 30 s followed by 35 cycles of 95 °C for 5 s, 60 °C for 30 s. The fluorescent signal of the amplified products was detected during the last step of each cycle. Melting curve analysis was performed after amplification to distinguish the targeted PCR product from non-targeted product. The melting curves were obtained by slow heating the samples from 65 °C to 95 °C at a rate 0.5 °C/s.

### 2.7. Isolation and Identification of Bacteria from Feces

Fecal samples were diluted with phosphate-buffered saline (PBS) and applied to Brain Heart Infusion agar (BD, Franklin Lakes, NJ, Japan) and modified Gifu Anaerobic Medium agar (Nissui, Tokyo, Japan) and cultured under anaerobic conditions. After 24 h to 72 h culture, some colonies were picked and the isolated colonies lysed in 100 µL of 50 mM NaOH. After incubation at 95 °C for 10 min, the lysed colonies were centrifuged at 3000× *g* for 10 min. Then, 20 µL of extracted solution was diluted with 100 µL Tris–HCl (pH 7.0-7.2). The diluted DNA was used as template for PCR amplification. The PCR reaction mixtures (25 µL) consisted of 18 µL of KOD FX Neo reagent solution (12.5 µL 2× buffer, 5 µL dNTPs, and 0.5 µL enzyme solution), 0.75 µL of each primer (10 µM), 2 µL of DNA template, and 3.5 µL of distilled water. The amplification profile consisted of one cycle at 94 °C for 2 min followed by 40 cycles of 98 °C for 10 s, 55 °C for 30 s, and 68 °C for 30 s with a final extension step of 72 °C for 5 min. After purification using ExoSAP-IT ExpressPCR Cleanup Reagents (ThermoFisher, Waltham, MA, Japan), the samples were sequenced using capillary sequence services from Hokkaido System Science Co., Ltd. (Sapporo, Hokkaido, Japan).

### 2.8. Evaluation of Sorbitol Degradation and Measurement of Sorbitol Concentration in Culture Supernatant

M9 medium [17] was used as a control medium to culture Enterobacteriaceae isolates. The other bacteria were cultured using the Gifu Anaerobic Medium (GAM) Semisolid without Dextrose (Nissui), prepared in water and filtered to remove the agar. When sugar was added to the media, the concentration was adjusted to 10 mg/mL. Following incubation under anaerobic conditions, 10 µL of the bacterial culture broth from the previous day was placed in each medium. Bacterial growth was evaluated based on absorbance at 600 nm or 570 nm that was measured using an Infinite 200 Pro plate reader (Tecan, Seestrasse, Switzerland). Sorbitol concentrations in the culture supernatants were measured using an EnzyChrom Sorbitol Assay Kit (BioAssay Systems, Hayward, CA, USA).

### 2.9. Statistical Analysis

Statistical analyses were performed using R (version 3.6.3) (R Foundation, Viennam, Austria). For parametrical data when the variances were equal, we used Student’s *t*-test for two groups, and one-way analysis of variance (ANOVA) followed by Dunnett’s test (post-hoc test) for more than two groups, respectively. When the variances were unequal, we used Welch’s *t*-test for two groups, and ANOVA followed by Steel’s test (post-hoc test) for more than two groups, respectively. Differences with *p*-values < 0.05 were considered statistically significant.

## 3. Results

### 3.1. Specific Gut Microbiota Played a Protective Role against Sorbitol-Induced Diarrhea

**Figure 1:**
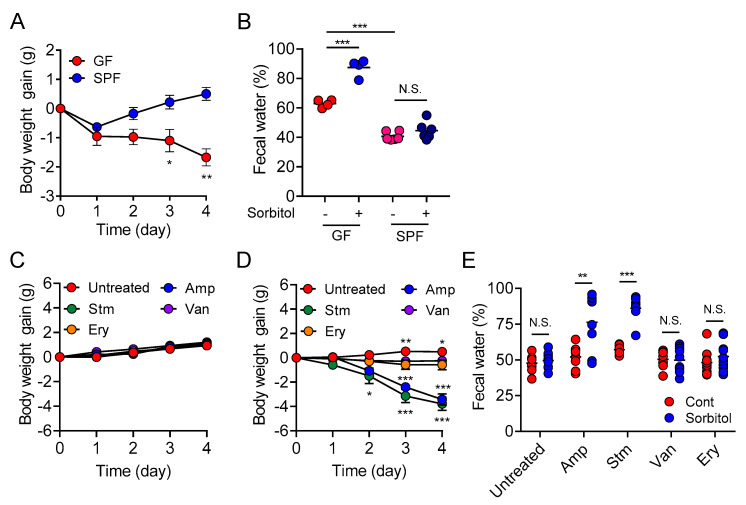
Specific Gut Microbiota Contributes to the Suppression of Sorbitol-Induced Diarrhea. (**A**,**B**) SPF and germ-free (GF) ICR mice were provided drinking water containing 5% sorbitol for 4 days. (**A**) Body weight changes from day 0 to day 4 (SPF, *n* = 6; GF, *n* = 4). Data are mean ± SEM. * *p* < 0.05, ** *p* < 0.01 compared to the day 0 weight of each group using Dunnett’s test. (**B**) Fecal water content at day 4. Each dot represents an individual mouse and the horizontal bars indicate mean values. N.S.; not significant, *** *p* < 0.001 by Tukey’s multiple comparisons test. SPF, specific pathogen-free; GF, germ-free. (**C**–**E**) The C57BL/6J mice were provided drinking water with or without 5% sorbitol for 4 days after antibiotic treatment as indicated. Cont, control; Sorbitol, 5% sorbitol; Amp, ampicillin; Stm, streptomycin; Van, vancomycin; Ery, erythromycin. (**C**) Body weight changes from day 0 to day 4 during treatment with antibiotics. Data are mean ± SEM. (**D**) Body weight changes from day 0 to day 4 during treatment with antibiotics and 5% sorbitol. Data are mean ± SEM. * *p* < 0.05, ** *p* < 0.01, *** *p* < 0.001 compared to the day 0 weight of each group using Dunnett’s test. (**E**) Fecal water content at day 0 and 4 after treatment with 5% sorbitol. Each dot represents an individual mouse and the horizontal bars indicate mean values. Data are from two independent experiments. N.S.; not significant, ** *p* < 0.01, *** *p* < 0.001 by Welch’s *t*-test.

We first investigated the role of gut microbiota in sorbitol-induced diarrhea by comparing susceptibility to sorbitol-induced diarrhea in specific pathogen–free (SPF) mice, which have an intact gut microbiota, and germ-free (GF) mice, which lack a gut microbiota. Unlike that for the SPF mice, the body weight of the GF mice receiving sorbitol continued to decrease over the four-day experimental period and significant weight loss was observed at four days compared with that of the SPF mice (Figure 1A). GF mice, but not SPF mice, had increased fecal water content four days after receiving sorbitol (Figure 1B). These results indicated the gut microbiota played a protective role against sorbitol-induced diarrhea.

We next investigated whether specific gut bacteria helped protect against sorbitol-induced diarrhea. To alter the composition of the gut microbiota, mice were treated with antibiotics that exhibit different antimicrobial spectra, and their susceptibility to sorbitol-induced diarrhea was examined. Treatment with antibiotics did not influence body weight change (Figure 1C). In the ampicillin-treated and streptomycin-treated mice, significant weight loss was observed after the administration of sorbitol compared with that in the antibiotic-free control group. These changes were not observed in the vancomycin-treated or erythromycin-treated mice (Figure 1D). Significant increases in sorbitol-induced fecal water content were observed in the ampicillin-treated and streptomycin-treated mice, but not in the vancomycin-treated or erythromycin-treated mice (Figure 1E). These results suggested vancomycin-resistant and erythromycin-resistant bacteria played protective roles against sorbitol-induced diarrhea. The involvement of gut bacteria in sugar alcohol-induced diarrhea other than sorbitol was confirmed based on mannitol-induced diarrhea, which was exacerbated by the administration of ampicillin (Figure S1). Overall, our results indicated that specific gut bacteria contributed to the inhibition of sugar alcohol-induced diarrhea. 

### 3.2. Enterobacteriaceae Were Identified as Gut Bacteria Responding to Sorbitol

**Figure 2:**
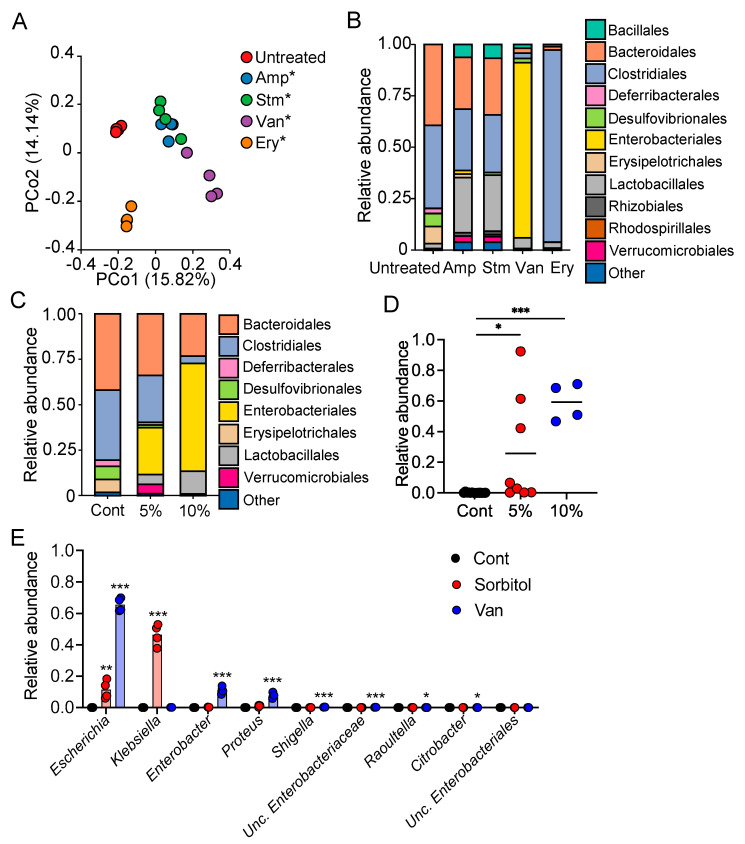
Identification of Enterobacteriaceae as Gut Bacteria Responding to Sorbitol. (**A**) Principal coordinate analysis (PCoA) plot generated using an unweighted UniFrac metric. Amp, ampicillin; Stm, streptomycin; Van, vancomycin; Ery, erythromycin. * *p* < 0.05 by PERMANOVA *t*-test. (**B**) Relative abundance of operational taxonomic units (OTUs) in fecal samples from C57BL/6J mice untreated or treated with antibiotics. The various colors correspond to each bacterial order indicated. Amp, ampicillin; Stm, streptomycin; Van, vancomycin; Ery, erythromycin. (**C**) Relative abundance of OTUs in fecal samples from sorbitol-treated C57BL/6J mice (*n* = 4). The various colors correspond to each bacterial order indicated. Cont, control; 5%, 5% sorbitol; 10%, 10% sorbitol. (**D**) Relative abundances of OTUs assigned to the family Enterobacteriaceae. Cont, control; 5%, 5% sorbitol; 10%, 10% sorbitol. Each dot represents an individual mouse and the horizontal bars indicate mean values. * *p* < 0.05, *** *p* < 0.001 compared with the control group using Dunnett’s *t*-test. (**E**) Relative abundances of OTUs assigned to the genera of the family Enterobacteriaceae. Bars in the graph are mean values. Each circle represents an individual mouse. * *p* < 0.05, ** *p* < 0.01, *** *p* < 0.001 compared to the Cont group using Steel’s test. Cont, control; Amp, ampicillin; Stm, streptomycin; Van, vancomycin; Ery, erythromycin.

To identify the gut bacteria contributing to the suppression of sorbitol-induced diarrhea, we evaluated the composition of the gut microbiota of mice treated with antibiotics by 16S rDNA analysis. The analysis confirmed that treatment with each of antibiotics altered the gut microbiota composition (Figure 2A), and revealed that bacteria of the orders Enterobacteriales and Clostridiales were the dominant bacteria in the vancomycin-treated and erythromycin-treated group, respectively (Figure 2B). Notably, the abundance of Enterobacteriales increased with the administration of increasing amounts of sorbitol in a concentration-dependent manner (Figure 2C,D). At the genus level, treatment with sorbitol and vancomycin resulted in the greatest increase in the abundance of *Escherichia, Klebsiella, Enterobacter*, and *Proteus* in the family Enterobacteriaceae (Figure 2E). In contrast, treatment with erythromycin increased the abundance of *Lachnoclostridium* in the family Clostridiaceae in the gut (Figure 2B and Figure S2). These results suggest that Enterobacteriaceae are one of the families of gut bacteria responding to sorbitol and may be involved in the suppression of sorbitol-induced diarrhea.

### 3.3. E. coli Degraded Sorbitol and Suppressed Sorbitol-Induced Diarrhea

**Figure 3:**
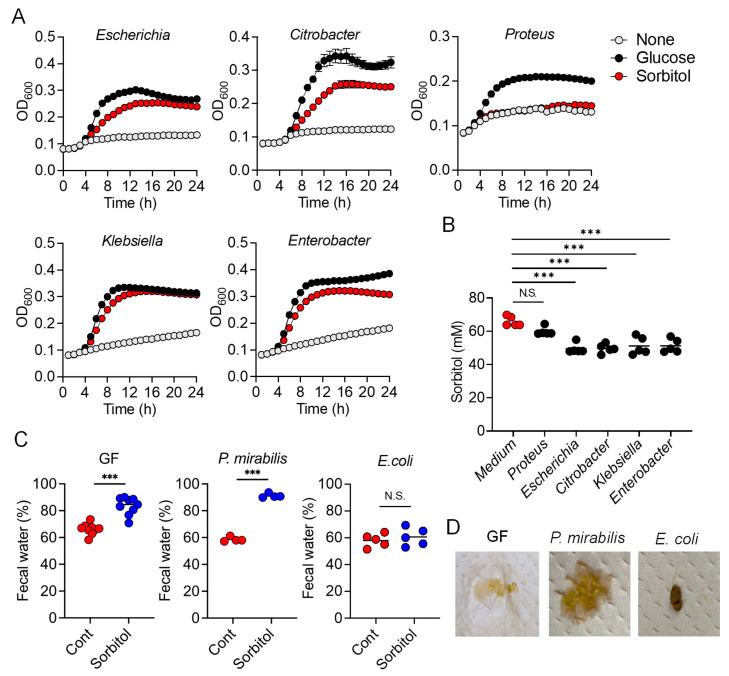
*Escherichia coli* are Able to Degrade Sorbitol and Suppress Sorbitol-Induced Diarrhea. (**A**) Each Enterobacteriaceae species was individually grown in M9 medium or M9 medium supplemented with 1% glucose or sorbitol and optical density (O.D.) at 600 nm was measured over time. (**B**) Sorbitol concentrations in 24 h culture supernatants of each Enterobacteriaceae (*n* = 5). Horizontal bars are mean values. N.S.; not significant, *** *p* < 0.001 compared with the control (Medium) group using Dunnett’s *t*-test. (**C**,**D**) IQI Germ-free (GF) mice or GF mice colonized with *E. coli* or *P. mirabilis* were provided drinking water containing 5% sorbitol for 4 days (GF mice, GF + *E. coli* mice; GF + *P. mirabilis* mice). (**C**) Fecal water content at day 0 and 4 after treatment with 5% sorbitol. Cont: control; 5%:5% sorbitol. Each dot represents an individual mouse and the horizontal bars indicate mean values. N.S.; not significant, *** *p* < 0.001 by Welch’s *t*-test. (**D**) Image of representative feces from the gnotobiotic mice given 5% sorbitol for 4 days.

To confirm that Enterobacteriaceae suppressed sorbitol-induced diarrhea by consuming sorbitol in the colon, we isolated Enterobacteriaceae bacteria which abundance was increased by sorbitol and/or vancomycin treatment, including *E. coli*, *Citrobacter farmeri*, *Klebsiella pneumoniae*, *Enterobacter* spp., and *Proteus mirabilis*, and assessed their ability to utilize sorbitol as a sole carbon source in culture medium. M9 media supplemented with 1% glucose or sorbitol were prepared and bacterial growth was measured based on absorbance. The growth of *E. coli*, *C. farmeri*, *K. pneumoniae*, and *Enterobacter* spp. was promoted by the addition of sorbitol to the media, like as glucose, but *P. mirabilis* failed to grow in sorbitol-supplemented medium (Figure 3A). This suggested that at least four types of bacteria from family Enterobacteriaceae were able to degrade sorbitol. Consistent with this, the concentration of sorbitol in the supernatants of the *E. coli*, *C. farmeri*, *K. pneumoniae*, and *Enterobacter* spp. cultures was lower than for control medium, but comparable in the supernatants of the *P. mirabilis* cultures. (Figure 3B). We also found that some *Clostridium* species (re-classified as *Lachnoclostridium* [18]) were able to utilize sorbitol and promoted their growth (Figure S3). 

We next conducted a gnotobiotic experiment using sorbitol-degrading bacteria and non- sorbitol-degrading bacteria in order to verify that the ability of Enterobacteriaceae to degrade sorbitol helped protect against sorbitol-induced diarrhea. GF mice were inoculated with *E. coli* as a sorbitol-degrading bacterium and *P. mirabilis* as a non-sorbitol-degrading bacterium. The mice were allowed access to drinking water containing sorbitol ad libitum. The fecal water content was significantly increased after administration of sorbitol in the GF mice and GF mice inoculated with *P. mirabilis*, while that in the GF mice inoculated with *E. coli* did not change when treated with sorbitol (Figure 3C). Representative results regarding the stool properties of each groups after sorbitol treatment are shown in Figure 3D. These results indicated that sorbitol-consuming members of Enterobacteriaceae prevented sorbitol-induced diarrhea.

### 3.4. SrlD Was Essential for Suppression of Sorbitol-Induced Diarrhea by E. coli

**Figure 4:**
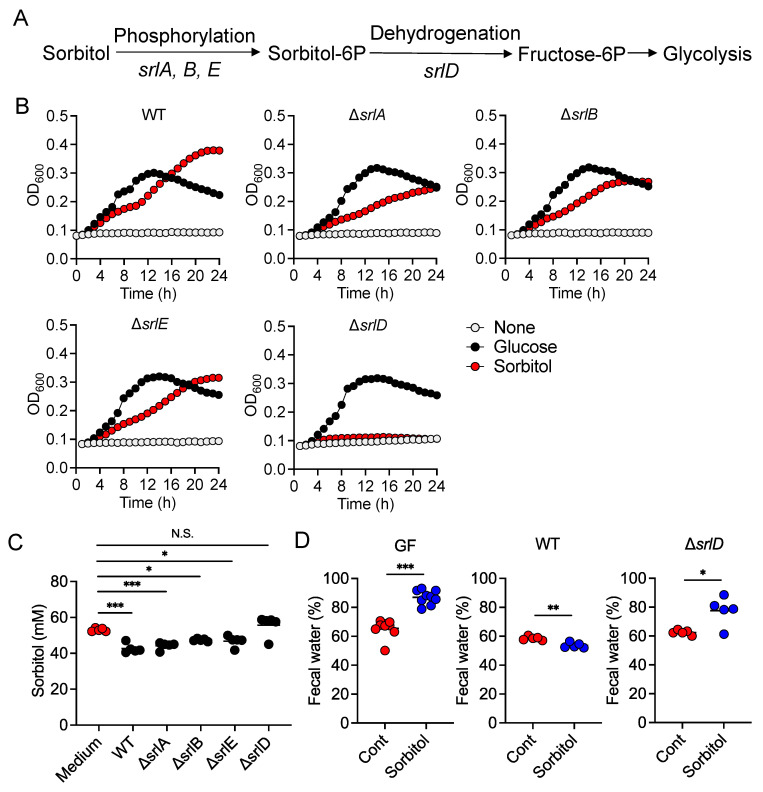
*srlD* Is Required for Suppression of Sorbitol-Induced Diarrhea by *Escherichia coli*. (**A**) The pathway of sorbitol metabolism. (**B**) WT and *srlA*, *srlB*, *srlE*, or *srlD* mutants of *E. coli* were grown in M9 medium or M9 medium supplemented with 1% glucose or sorbitol and optical density (O. D.) at 600 nm was measured over time. (**C**) Sorbitol concentration in 24 h culture supernatants of WT or mutant *E. coli* strains (*n* = 5). Horizontal bars are mean values. * *p* < 0.05, *** *p* < 0.001, compared with the control (Medium) group using Dunnett’s *t*-test. (**D**) ICR Germ-free (GF) mice or GF mice colonized with WT or *srlD* mutant of *E. coli* were provided drinking water containing 5% sorbitol for 4 days. Fecal water content at day 0 and 4 after treatment with 5% sorbitol was assessed. Each dot represents an individual mouse and the horizontal bars indicate mean values. * *p* < 0.05, ** *p* < 0.01, *** *p* < 0.001 by Welch’s *t*-test. WT, wild-type *E. coli*. N.S., not significant.

To identify the pathway responsible for metabolizing sorbitol in *E. coli*, we used mutants lacking *srlA*, *srlB*, and *srlE* genes encoding Enzyme II complex which is involved in sorbitol transport, including, along with *srlD*, the gene encoding PTS-dependent sorbitol-6-phosphate dehydrogenase which generates fructose 6-phosphate for glycolysis (Figure 4A). The *srlA*, *srlB*, and *srlE* mutants were able to grow in media containing either glucose or sorbitol as the sole carbon source at rates comparable to those of the wild-type control (Figure 4B). In contrast, the *srlD* mutant was able to grow in a medium containing glucose as a sole carbon source, but not in a medium containing sorbitol as a sole carbon source (Figure 4B). In addition, sorbitol concentrations in the supernatants of the wild-type and *srlA*, *srlB*, and *srlE* mutant strains were lower than that of the control medium (Figure 4C). Meanwhile, sorbitol concentration in the supernatants of the *srlD* mutant cultures was comparable to that of the control medium (Figure 4C). Consistent with this, the inoculation of GF mice with the wild-type *E. coli* protected the mice against sorbitol-induced diarrhea, but not with the *srlD* mutant (Figure 4D). These results suggested that sorbitol metabolism, mediated by the sugar phosphotransferase pathway in *E. coli*, contributed to the suppression of sorbitol-induced diarrhea.

## 4. Discussion

In the current study, we demonstrated the protective role of gut bacteria against sugar alcohol-induced diarrhea using both GF mice that lack gut bacteria and mice that had altered gut microbiota as a result treatment with antibiotics. When exposed to sorbitol at a dose that failed to induce diarrhea in SPF mice, GF mice exhibited severe diarrhea and significant weight loss. GF mice are known to have higher fecal water content than that of SPF mice under normal conditions, but while the fecal water content of the SPF mice in our study did not change with sorbitol treatment, the fecal water content of the GF mice increased with sorbitol treatment. Since antibiotics such as ampicillin, streptomycin, vancomycin, and erythromycin have different antimicrobial spectra, administration of those antibiotics changed the gut microbiota composition of mice to specific states that were dependent on their antimicrobial spectra. Mice with gut microbiota composition altered by ampicillin and streptomycin treatments exhibited increased susceptibility to sorbitol-induced diarrhea. In comparison, mice with gut microbiota composition that had increased proportions of Enterobacteriaceae and *Lachnoclostridium* as a result of vancomycin and erythromycin treatment, respectively, did not show an increase in their susceptibility to sorbitol-induced diarrhea. Interestingly, the abundance of Enterobacteriaceae increased in a concentration-dependent manner as sorbitol intake increased. This effect was particularly dramatic in mice that received 10% sorbitol. This sorbitol-induced growth of Enterobacteriaceae most likely contributed to the transient diarrhea and subsequent disappearance of diarrheal symptoms observed in these mice. Enterobacteriaceae expansion may also represent the mechanism underlying the acquisition of sorbitol tolerance observed in individuals after long-term intake.

At the species level, *E. coli* constituted approximately 70% of the gut Enterobacteriaceae population in vancomycin-treated mice with *Enterobacter* spp. and *P. mirabilis* accounting for the remaining 30%. As *Enterobacter* spp. and *P. mirabilis* were not able to utilize sorbitol as an energy source, *E. coli* appears to have been the main contributor to the suppression of sorbitol-induced diarrhea by Enterobacteriaceae. Indeed, the inoculation of GF mice with *E. coli* alone was sufficient to generate resistance to sorbitol-induced diarrhea.

While the pathways of sorbitol metabolism vary depending on the particular bacterial species, the sugar phosphotransferase system (PTS) is one of the major known pathways. Sorbitol is phosphorylated in this pathway to sorbitol-6-phosphate by a sugar-specific permease, which is a membrane-bound complex known as enzyme II. The sorbitol-6-phosphate is subsequently converted to fructose by PTS-dependent dehydrogenase and the fructose is used for glycolysis [19,20,21]. An *E. coli* mutant lacking *srlD*, which encodes the PTS-dependent sorbitol-6-phosphate dehydrogenase, was unable to degrade sorbitol and did not protect mice against sorbitol-induced diarrhea. This indicates the sorbitol degradation by *E. coli* was dependent on PTS. However, mutants of *srlA*, *srlB*, or *srlE*, which are genes encoding components of the enzyme II complex, were able to utilize sorbitol as an energy source, suggesting that an enzyme II complex–independent pathway for the production of sorbitol-6-phosphate via phosphorylation of sorbitol may exist in *E. coli.* On the other hand, we also showed that some *Clostridium* species (re-classified as *Lachnoclostridium*) were able to utilize sorbitol. However, *Clostridium* is known to be able to directly metabolize sorbitol to fructose unlike *E. coli* which metabolizes sorbitol to fructose via the PTS pathway [22,23].

Although some strains of *E. coli* can cause infectious and inflammatory diseases in humans, many others live peacefully in human intestines, aiding in digestion and even defending their host against harmful microbes [24]. We found that *E. coli* was increased and suppressed sorbitol-induced diarrhea after the ingestion of sorbitol. In human adults, *E. coli* is not the dominant bacteria in the intestine. However, these microbes may maintain a hospitable environment in the gut by preventing the detrimental effects of artificial sweeteners which are widely used in processed foods.

Our current study revealed the relationship between sugar alcohol-induced diarrhea and the gut microbiota, and identified specific gut bacteria that responded to and degraded the sorbitol. However, mannitol and xylitol (which like sorbitol, are poorly-absorbed sugar alcohols) are also widely used as artificial sweeteners in processed foods. As the metabolism of these sugar alcohols by the gut bacteria may differ from that of sorbitol, the species of gut bacteria that protect against diarrhea induced by these sugar alcohols may also be different. Further work is needed to clarify whether sugar alcohols other than sorbitol are degraded by different gut microbes.

The gut microbiota has been reported to be associated with various diseases, including some lifestyle-related diseases [25,26,27]. Accordingly, manipulation of the gut microbiota has received attention for its potential exploitation in supporting human health, such as the use of stool transplantation or metabolites derived from gut microbiota [28,29]. Prebiotics are compounds in food that induce the growth or activity of beneficial microorganisms, such as by altering the composition of the gut microbiome [30]. In contrast, dysbiosis is a term for microbial imbalance that may causes negative health symptoms [31]. Dysbiosis can arise from diverse causes, including antibiotic use or consumption of an inappropriate diet [32]. Our current findings regarding the effect of sorbitol on the gut microbiota suggest that artificial sweeteners in processed foods can act as either prebiotics that promote a healthy gut or as inappropriate dietary elements that may cause dysbiosis. We believe our findings provide helpful information elucidating the effects of artificial sweeteners in processed foods on human health via gut microbiota.

## Data Availability

The data presented in this study are available on request from the corresponding author.

## Supplementary Materials

The following are available online at https://www.mdpi.com/article/10.3390/nu13062029/s1, Figure S1. Mannitol (Man)-Induced Diarrhea is Induced by Ampicillin (Amp) Administration; Figure S2. Relative Abundance of Genera of the Order Clostridiales; Figure S3. Some Clostridium species (re-classified as Lachnoclostridium) Are Able to Utilize Sorbitol as a Carbon Source.
