# Supplementary material for: Gut Microbiota Prevents Sugar Alcohol-Induced Diarrhea
Source: Nutrients. 2021 Jun 12;13(6):2029. doi: 10.3390/nu13062029 (PMC8231616; doi:10.3390/nu13062029)
Supplement: Supplementary file 1 [file nutrients-13-02029-s001.zip › nutrients-1222227-supplementary.pdf]

Supplemental Information

Gut Microbiota Prevents Sugar Alcohol-induced Diarrhea

Kouya Hattori, Masahiro Akiyama, Natsumi Seki, Kyosuke Yakabe, Koji Hase, Yun-Gi Kim

Figure S1

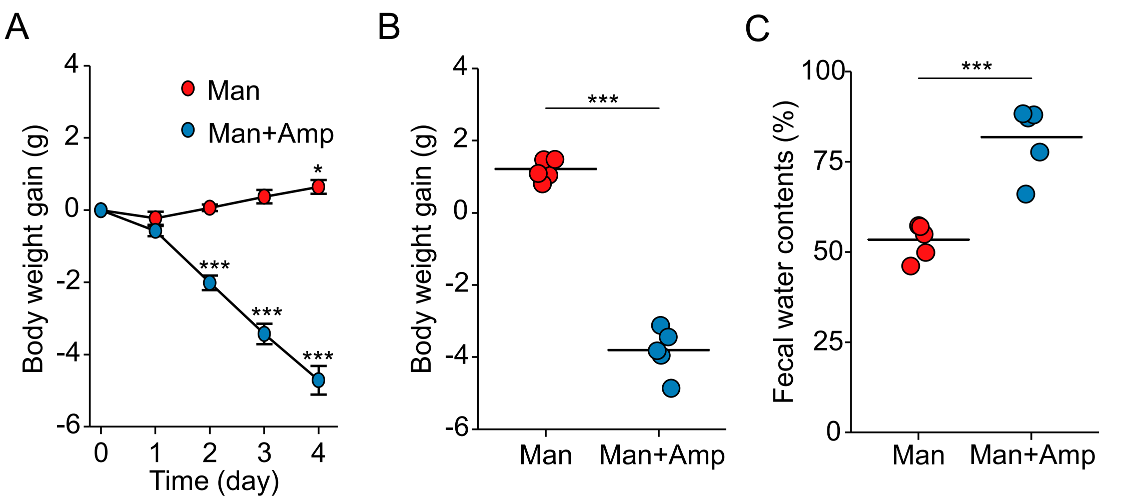

Figure S1. Mannitol (Man)-Induced Diarrhea is Induced by Ampicillin (Amp) Administration.

(A) Body weight changes from day 0 to day 4. Data are mean ± SEM. *$p < 0.05$, ***$p < 0.001$ compared to the day 0 weight of each group using Dunnett's test. (B) Body weight gain and (C) fecal water contents at day 4. Each dot represents an individual mouse and the horizontal bars indicate mean values. ***$p < 0.001$ by Student's t-test.

Figure S2

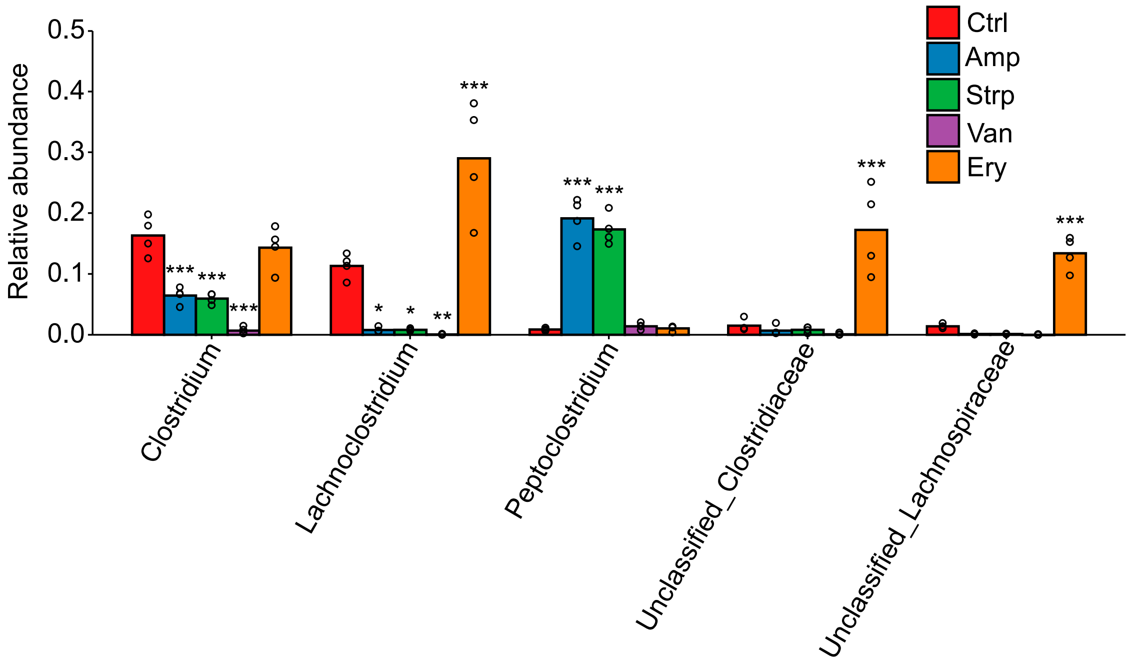

Figure S2. Relative Abundance of Genera of the Order Clostridiales.

Relative abundances of operational taxonomic units (OTUs) assigned to genera of the order Clostridiales. Bar charts are mean values. Each circle represents an individual mouse. $*p < 0.05$, $**p < 0.01$, $***p < 0.001$, as compared with the Ctrl group using Dunnett’s t-test.

Figure S3

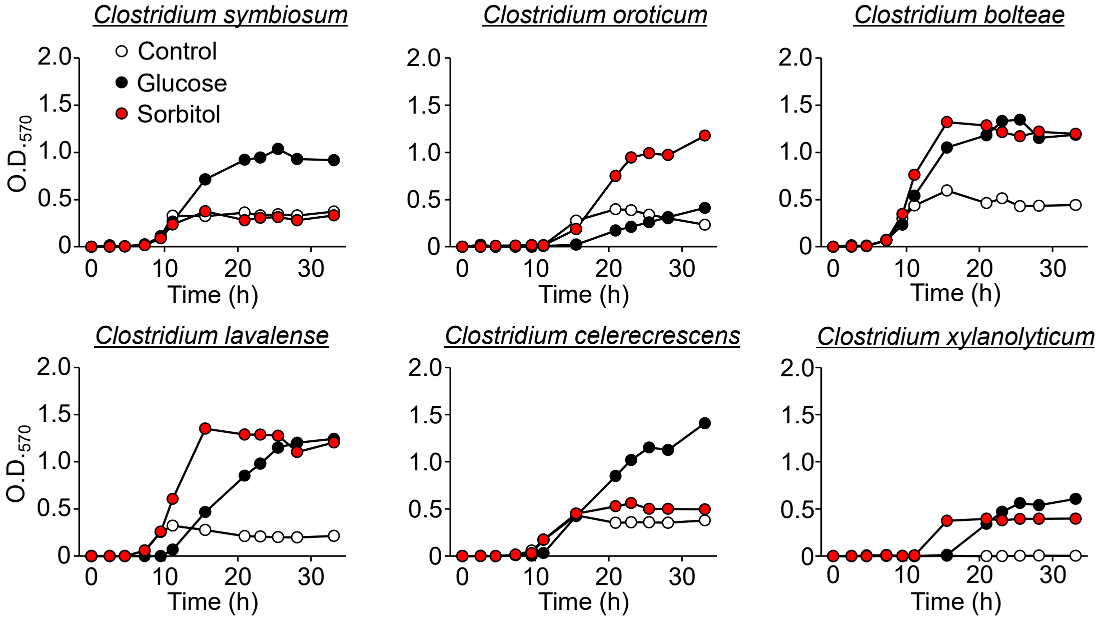

Figure S3. Some *Clostridium* species (re-classified as *Lachnoclostridium*) Are Able to Utilize Sorbitol as a Carbon Source.

Growth of each *Lachnoclostridium* strain evaluated using changes in optical density (O.D.) at 570 nm. Three types of media were prepared (control, glucose supplemented, and sorbitol supplemented).
